# Supplementary material for: Deep vision-based real-time hand gesture recognition: a review
Source: PeerJ Comput Sci. 2025 Jun 24;11:e2921. doi: 10.7717/peerj-cs.2921 (PMC12453657; doi:10.7717/peerj-cs.2921)
Supplement: Supplemental Information 1 [file peerj-cs-11-2921-s001.docx]

**Supplementary Figures for Research Questions**

# **RQ1: What type of models and procedures are used in the real time HGR through deep learning?**

In accordance with Table 7, Figure 5 illustrates the distribution of various underlying models, each represented by a unique color and numerical value. Moreover, the overlapping parts of the colors are the combination of two or more underlying models.


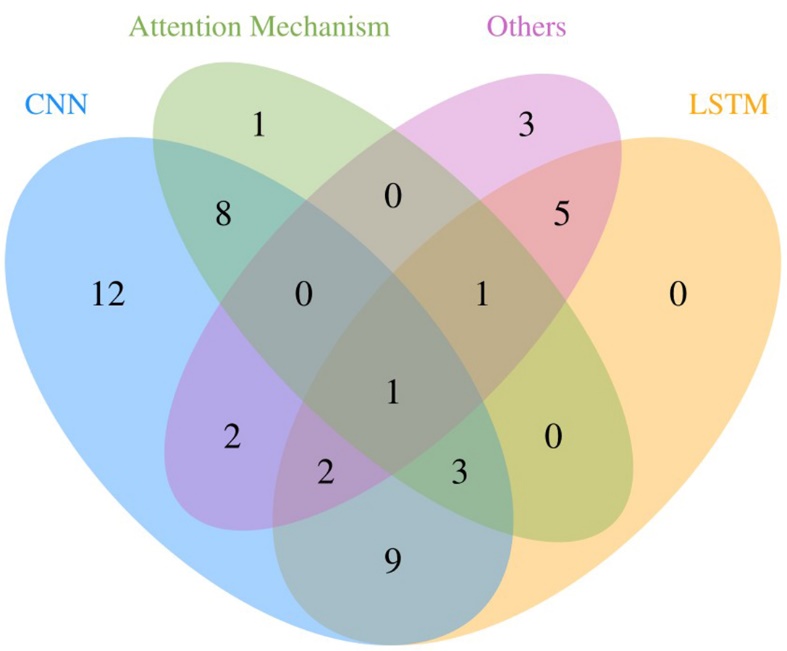


Figure 1 The distribution of underlying models in methods shows the number of CNN, LSTM, attention mechanism, and others and their combination. Among them, CNN has the highest frequency of occurrence, followed by multiple models composite.


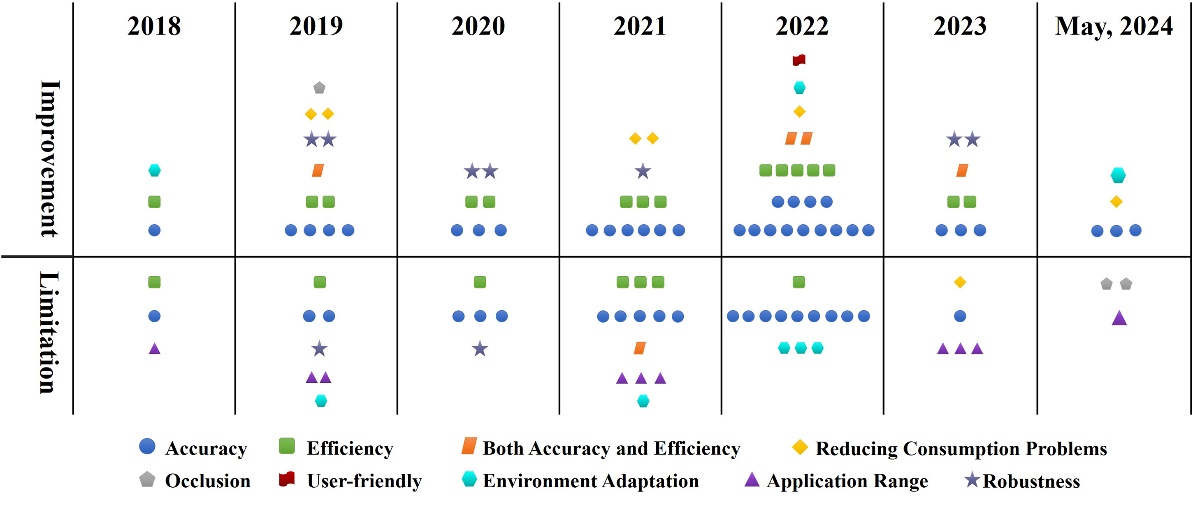


Figure 2 Improvements and limitations in the performance of the models

# **RQ2: What are the performance metrics used to evaluate the HGR models?**


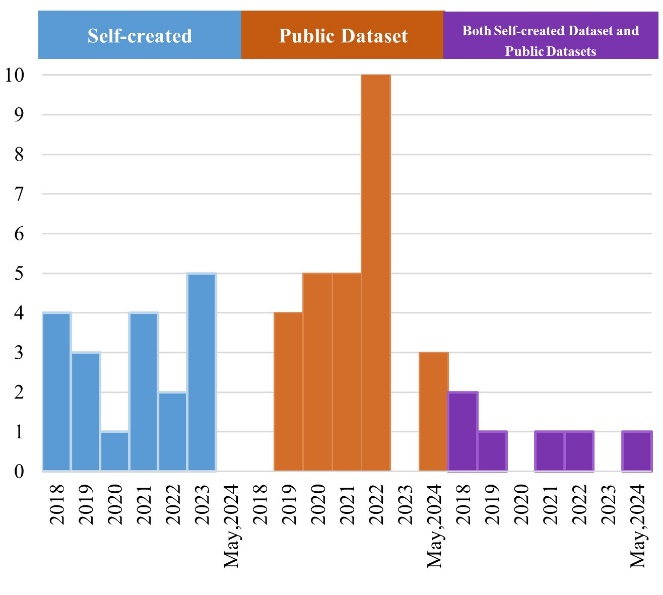


Figure 3 Analysis of the datasets chosen for training.


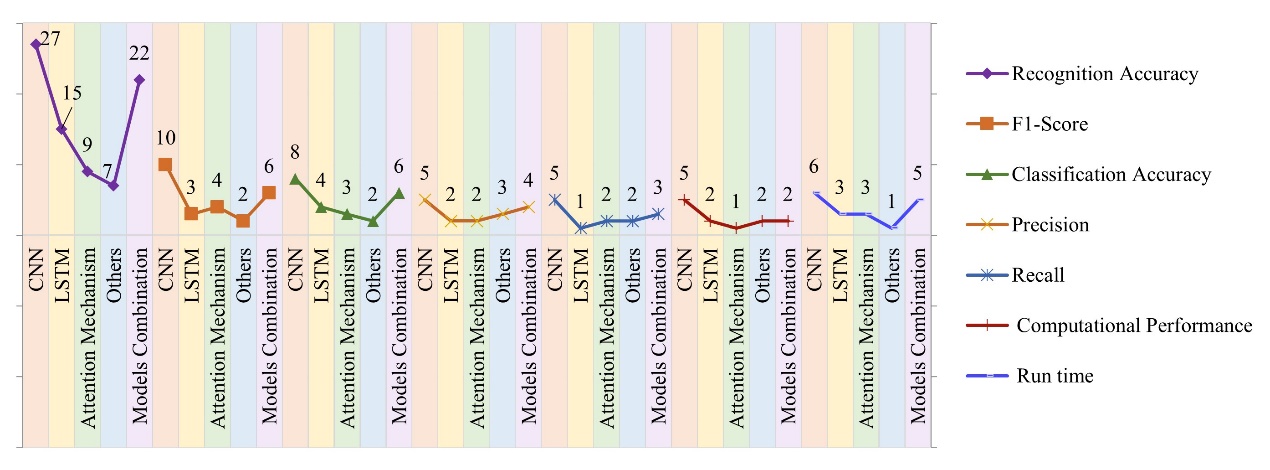


Figure 4 The relationship between evaluation parameters choosing and underlying models. The CNN and underlying models combination are higher than others in each evaluation parameter.


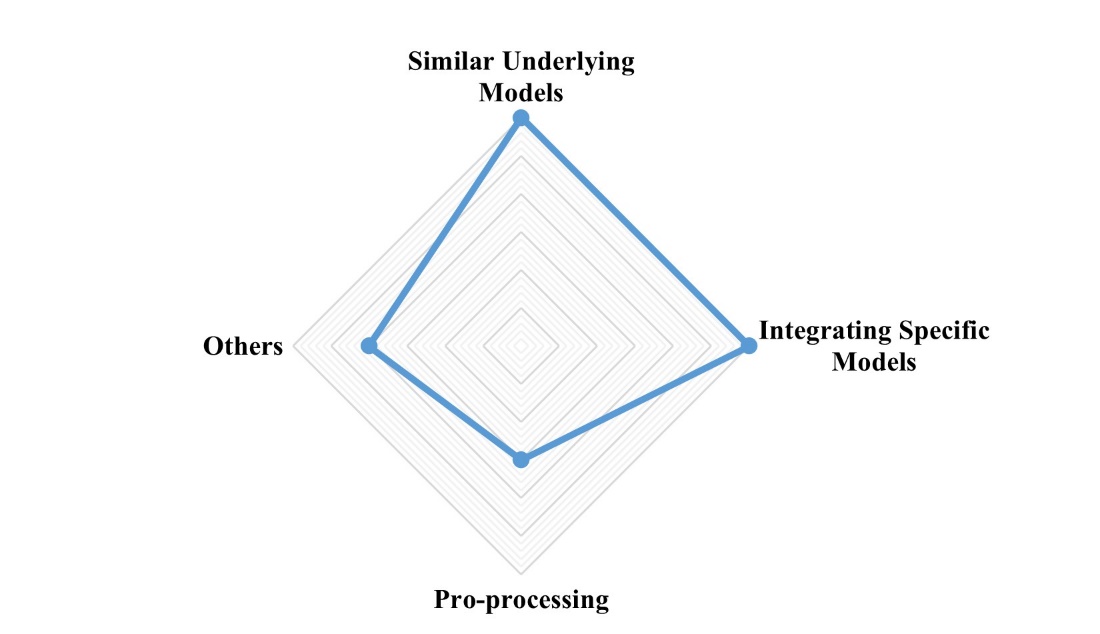


Figure 5 Ablation study. Among them, both similar underlying models and integrating specific models are the largest.

# **RQ3: Which research gap remains in real-time HGR using deep learning?**


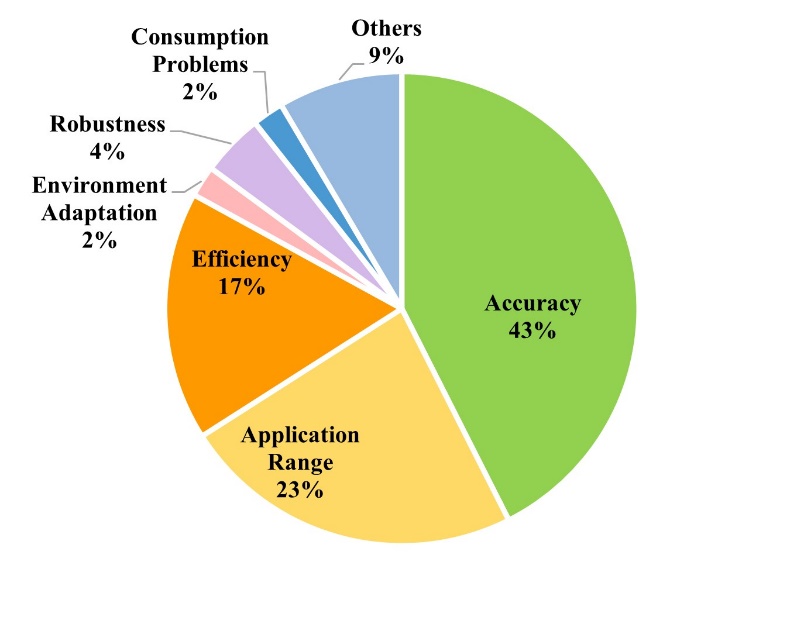


Figure 6 The proportion of different limitations.
